# Well-being and quality of life of Kenyan nurses, midwives and community health volunteers: Measurement properties and correlates of the BBQ and WHO-5

**DOI:** 10.1371/journal.pgph.0005510

**Published:** 2025-12-02

**Authors:** Paul Mwangi, Sabina Adhiambo Odero, Rachel Odhiambo, Brenda Mumbua Nzioka, Constance Sibongile Shumba, Eunice Ndirangu-Mugo, Amina Abubakar

**Affiliations:** 1 Institute for Human Development, Aga Khan University, Nairobi, Kenya; 2 Department of Clinical, Neuro- and Developmental Psychology, Faculty of Behavioural and Movement Sciences, Vrije Universiteit Amsterdam, Amsterdam, The Netherlands; 3 School of Nursing and Midwifery, East Africa, Aga Khan University, Nairobi, Kenya; 4 Division of Epidemiology and Social Sciences, Institute for Health and Humanity, Medical College of Wisconsin, Milwaukee, Wisconsin, United States of America; 5 Neurosciences Unit, Kenya Medical Research Institute (KEMRI)-Wellcome Trust Research Programme, Centre for Geographic Medicine Research, Kilifi, Kenya; PLOS: Public Library of Science, UNITED STATES OF AMERICA

## Abstract

Healthcare workers’ quality of life (QoL) and well-being is directly linked to the quality of services they deliver. However, there is a shortage of validated measures to assess QoL, well-being and their predictors among healthcare workers in Kenya, as in other LMICs. This study aimed to assess the reliability and validity of the Brunnsviken Brief Quality of Life Scale (BBQ) and WHO-5 well-being index (WHO-5) among nurses, midwives and community health volunteers (CHVs) in Kenya. The BBQ and WHO-5 were selected due to their established psychometric strengths, brevity, and prior use in healthcare settings. Additionally, the sociodemographic, psychological, and health system factors associated with QoL and well-being in this population were also examined. This was a national, cross-sectional study conducted among 1907 nurses/midwives and 2027 CHVs in Kenya. Data was collected from June to November 2021 through telephone interviews. Internal consistency of the BBQ and WHO-5 scales was evaluated using Cronbach’s alpha and Macdonald’s omega. The BBQ and WHO-5 scales’ construct validity were evaluated using Confirmatory Factor Analysis (CFA). Correlation and invariance analysis were used to evaluate divergent validity and measurement invariance, respectively. Univariate and adjusted linear regression models were used to explore the sociodemographic, psychological, and health system factors associated with QoL and well-being among the healthcare workers. The BBQ and WHO-5 had adequate reliability (both alpha and omega >0.70). CFA supported unidimensional structures of both the BBQ and the WHO-5 with acceptable model fit indices. Divergent validity and measurement invariance across language of administration (English and Swahili) were also established. Our results also showed that multiple individual and work environment factors influence both well-being and QoL of Kenyan healthcare workers. Therefore, BBQ and WHO-5 are reliable and valid measures for use among Kenyan healthcare workers. Since workplace and personal factors influence QoL and well-being of healthcare workers, multi-pronged approaches and interventions could be useful in improving the well-being and QoL of healthcare workers in Kenya.

## Introduction

Quality of life (QoL) is a multifaceted construct that typically includes subjective assessment of both advantageous and disadvantageous aspects of one’s life, such as health and socioeconomic status [1–3]. According to Katschnig [4], subjective QoL is a broad concept that can be defined as an overall, self-perceived life satisfaction in important life areas such as work, friendship, leisure, and the ability to be creative and learn new things. On the other hand, well-being refers to feeling content with life, being healthy, having emotional resilience and a sense of purpose or meaning in one’s life [5]. Noteworthy, an individual can experience well-being even while managing mental or physical health challenges [6]. Although quality of life and well-being are closely related, they should be treated as separate concepts [7,8]. Notably, subjective QoL focuses on various life domains, while well-being focuses on individuals’ psychological and emotional experiences [9,10].

QoL and well-being are important factors in healthcare workers’ productivity and ability to provide health services. They contribute to the healthcare workers commitment at work, attitude toward their patients, service to patients, efficiency, and overall provision of quality care leading to better patient outcomes [11]. Contrarily, poor QoL leads to absenteeism and poor-quality services affecting patient outcomes [11,12]. It is, therefore, important to assess and devise strategies to improve healthcare workers’ QoL and well-being.

Most studies tend to focus on QoL and well-being of patients, with few studies focusing on QoL and well-being among healthcare workers [11,13]. Working in a health care setting has been linked to high work-related stress and low QoL and well-being among nurses [14–16]. Factors such as increased workload, longer working hours, financial constraints, and high work-related stress have all been seen to be significant contributing factors [17–19]**.** However, there are a few studies published from sub-Saharan Africa (SSA) assessing the well-being and QoL of healthcare workers [20,21]. Partly, this could be due to a lack of funding and a paucity of validated psychological measures. Additionally, the available studies on healthcare workers focus on professional cadres like nurses and doctors while leaving out the non-professional cadres like community health workers. This is despite the contributions of these cadres in the health system specifically the provision of public health and/or social services and linkages to healthcare for the communities they serve, a task that has been shown to similarly expose them to psychological distress as professional cadres [22]. In this study, we included participants from both professional cadres (nurses and midwives) and a non-professional cadre (community health volunteers).

There have been many QoL instruments developed over the years. A systematic review of quality-of-life questionnaires developed and used in studies among adults between 2008 and 2018, revealed a total of 23 instruments available [23]. Some of these are quite long and would take time to administer effectively. However, there are also brief and simple quality of life and well-being scales that are readily available and do not require a license or have other restrictions on translation. These instruments aim to provide a convenient and efficient way to assess individuals’ QoL and well-being, allowing for broader usage and accessibility in research and clinical settings. Some of these tools include the Brunnsviken Brief Quality of Life Scale (BBQ) and the World Health Organization-Five (WHO-5) Well-being Index [2,24].

The Brunnsviken Brief Quality of Life Scale (BBQ) was designed to assess an individual’s subjective quality of life in six areas: recreation, philosophy of life, creativity, learning, self-esteem, and friendship [24]. The BBQ is a one-dimensional tool that is easily accessible, brief and has good psychometric properties [24,25]. Since its development, the tool has been translated into 30 different languages [26]. The BBQ has been used to assess the quality of life in various populations, including young adults, nursing students, older adults, and gay and bisexual men living in Australia and New Zealand [27–29].

The WHO-5 well-being index is a brief 5-item tool that measures psychological well-being in research and clinical setting [30]. The WHO-5 scale focuses on a person’s subjective well-being, including their mood (happiness, good spirits, calm, and relaxation), agility (being active, vigorous, waking up feeling fresh and rested), and general interest in the sense that their daily lives are filled with exciting things [31]. The WHO-5 scale has been translated into more than 30 languages worldwide since its development in 1998 [31]. Numerous studies have found that the WHO-5 scale is reliable and valid across various populations, including people living with type 1 diabetes, college students, adults, people with disabilities, and healthcare professionals [32–36]. However, the BBQ and WHO-5 scales have not been validated among healthcare workers in low- and middle-income countries (LMICs).

This study aimed to assess the reliability and validity of the BBQ and the WHO-5 scales among healthcare workers (nurses, midwives, and community health volunteers (CHVs)) across 47 counties in Kenya. Our analysis reports explicitly on the internal consistency, construct validity, measurement invariance, and divergent validity of the BBQ and WHO-5 well-being scales among nurses, midwives and CHVs, respectively. Additionally, we assessed the socio-demographic characteristics, psychological factors and health system measures associated with QoL and well-being.

## Methods

### Ethics statement

This study received ethical approval from the Aga Khan University Institutional Scientific and Ethics Review Committee (ISERC) for Kenya (IERC number 2021/IERC-32) and a research permit from National Commission for Science, Technology and Innovation (NACOSTI) license number NACOSTI/P/21/10034. Since this was a telephonic survey, all participants gave verbal informed consent before data collection. The verbal consent over the phone was audio recorded and kept as proof of consent. The Research Assistants signed a physical copy of the consent form as proof that they followed due process in obtaining the consent.

### Study setting, sample and sampling procedures

This was a nationwide cross-sectional study and conducted across 47 counties in Kenya. Study participants were nurses, midwives, and CHVs. The data collection was conducted between 1st June to 15^th^ November 2021. This study was part of a larger study that focused on mental health, psychological distress, and resilience among healthcare workers in Kenya during the COVID-19 pandemic. Detailed description of the samples and sampling procedures of the primary study are presented elsewhere [37]. Briefly, nurses/midwives were invited to participate in the study through the national registration and licensing body for nurses and midwives in Kenya – the Nursing Council of Kenya (NCK). For CHVs, we coordinated with community health focal persons in every county to identify and invite CHVs to take part in the study. Potential participants were included in the study if they were older than 18 years in age, had worked for more than 6 months, spoke English or Swahili, and consented to participate in the study.

### Data collection

The tools were administered over the phone to CHVs, nurses/midwives by trained research assistants and supervised by a research officer. Data collection for this study was carried out between June and November 2021 – a period characterized by surging COVID-19 case numbers and vaccination efforts. Travel and social interaction across the country was still limited, therefore we opted to carry out telephone interviews for the protection of the study team and participants. All telephone calls were made from a central location in Nairobi County, where the research institution is located. Since all data collection activities were conducted on phone, for all the questions, the research assistants repeatedly read out the potential answers for the participants to choose from, making the interviews last longer than if interviews had been face-to-face. The research assistants were recruited from healthcare allied fields such as public health, social work, etc., but were not attached to healthcare facilities at the time of data collection. They were all trained on the study specific protocols before data collection commenced. Since the tools used in this study were standardized measures, they administered them in the same manner to all the participants. Additionally, the research assistants received training on psychological first aid to be able to provide immediate support to participants who exhibited signs of distress at the point of data collection. Finally, we engaged independent counsellors to whom we referred participants as needed. The data sets were collected using the Open Data Kit (ODK) and stored on password-protected and secure Aga Khan University laptops.

### Data collection tools relevant to the present analysis

#### Dependent variables.

*Quality of life*: Respondents’ were asked their view on the importance of each of the six life areas in the BBQ, as well as their current satisfaction with it, totaling to 12 questions [24]. The items are scored on a 5-point Likert scale from 0 = strongly disagree to 4 = strongly disagree. The BBQ total score was obtained by multiplying the satisfaction and importance rating for each life area and then by summing the six products. The total score ranged from 0 to 96 with the highest score indicating the better QoL.

*Well-being:* The WHO-5 Well-being index was used to measure the well-being consisting of 5 items rated on a Likert scale of “0” (at no time) and “5” (all the time). The total score was obtained by summing the answers of five items. The total score ranged from 0 to 25, where the higher score indicates good well-being.

#### Independent variables.

*Socio-demographic characteristics:* A researcher-developed social demographic questionnaire was used to collect information about the participants’ sex, age, level of education, marital status, religion, psychosocial support from religion, work duration, income, and COVID-19-related factors (such as a change in working hours during COVID-19, exposure and testing positive to COVID-19).

*General anxiety*: The 7-item Generalized Anxiety Disorder scale (GAD-7) a screening tool was administered to examine the presence of anxiety symptoms. The scale consists of seven items with Likert scale scores of “0” (not at all) to 3 (nearly every day). The total score is 0–21, with 0–4, 5–9, 10–15, and >15 representing none, mild, moderate, and moderate to severe anxiety symptoms, respectively. In this study, the GAD-7 was administered to all participants, regardless of their known anxiety status, to enable screening anxiety symptoms.

*Depression*: Depressive symptoms were evaluated using the 9-item Patient Health Questionnaire (PHQ-9), a screening tool. The scale has nine items scored on a Likert scale ranging from 0 = Never to 3 = Almost every day. The raw scores are then added to form a PHQ-9 score ranging from 0 to 27, with higher scores suggesting severe depression symptoms (0–4 = None, 5–9 = Mild, 10–14 = Moderate, > 15 severe). All participants completed the PHQ-9 regardless of prior depression status, enabling screening for depressive symptoms across the study population.

*Perceived stress:* The Perceived Stress Scale (PSS-10) was administered to collect data on perceived stress [38]. It is a 10-item scale focusing on an individual’s feelings and thoughts over the last month. The items are scored on a five-point scale from 0 = never to 4 = very often. The PSS total score was obtained by summing all the items, and a high score indicated high perceived stress.

*Burnout*: The Oldenburg burnout inventory (OLBI) [39] was administered to this population. The tool has 16 items focusing on two dimensions: exhaustion and disengagement at the workplace; however, some items measure vigor and engagement, and these items are reverse scored when calculating the total OLBI score. The items are scored on a four-point Likert scale from 1 = strongly agree to 4 = strongly-disagree. The burnout score was generated by summing all items, and a high score indicated a high level of burnout.

*Stigma*: The 18 items Healthcare Workers Stigmatization Survey was used for assessing stigma in this population. This was scale was adapted from the stigma scale developed by Park, Lee [40] during the Middle East Respiratory Syndrome coronavirus (MERS-CoV) outbreak in South Korea. The scale is a 5-point Likert scored from 1 = Strongly Disagree to 5 = Strongly Agree. All 18 items were summed where the higher scores indicated higher levels of experienced stigma during COVID-19.

*Resilience:* The resilience of healthcare workers was assessed using the 6-item Brief Resilience Scale (BRS), which measures the perceived ability to recover or bounce back from stress [41]. The scale is scored on a 5-point Likert scale 1 = strongly disagree to 5 = strongly agree, where items 1, 3, and 5 are positively worded, and items 2, 4, and 6 are negatively worded. The negatively worded items were reverse scored, all items were summed and divided by 6 to generate the resilience score, where the higher average score indicated a higher perceived level of resilience.

*Social support:* The Multidimensional Scale of Perceived Social Support (MSPSS) was used to measure the healthcare workers’ perception of support from family, friends, and significant others [42]. The tool has 12 items, and responses range from 1 = “very strongly disagree” to 7 = “very strongly agree”. The items are summed to generate the MSPSS score, where a high score indicates a high level of social support.

*Work engagement:* The work engagement of healthcare workers was assessed using the Utrecht Work Engagement Scale-9 (UWES-9), which focuses on a positive work-related state of fulfilment characterized by vigor (items 1,2,5), dedication (items 3,4,7), and absorption (items 6,8,9) [43]. The scale is a 7-point Likert scale scored 0 = never to 6 = every day, and all items are summed together to generate the work engagement score. The higher the score, the higher the work engagement.

*Health system measure:* Constance Sibongile Shumba (CSS) developed the health system assessment scale comprising six domains during the COVID-19 pandemic. 1) *Individual capacity and motivation*: This domain assessed how frontline healthcare workers felt appreciated for their work, available to work, willing to work extra hours if needed, and proud and committed to their work. 2) *Job factors*: This domain assessed frontline healthcare workers’ workload management, balancing work demands and having enough time to rest. 3) *Workplace environment*: it assessed the availability of personal protective equipment (such as masks, sanitizer, handwashing facilities, infrared thermometers, and face shields), promotion of self-care/well-being, and access to information on coping mechanisms or stress management. 4) *Management and leadership*: this domain assessed frontline healthcare workers’ supervisors’ provision of guidance, involvement in planning, and communication. 5) *Structural support domain* assessed how frontline healthcare workers received psychosocial support. 6) *Health system capacity perceptions*: it assessed how frontline healthcare workers perceived the health system’s ability to increase resources, provide psychosocial support, and adhere to all proposed COVID-19 risk management measures. All items in each domain were 5-Likert scored from 1 (Strongly Disagree) to 5 (Strongly Agree), and all items in each domain were summed to generate a total score for each domain, with the higher score indicating better health system measures.

### Translation of the tools

All tools were forward-translated from English to Swahili by independent translators, and back-translated by the field team who were fluent in both English and Swahili. Furthermore, the field team harmonized the tools after forward and back translation and reviewed them all to ensure that they were culturally appropriate. This included rewording the items to begin with “You” instead of “I”, since the participants were interviewed via phone calls.

### Statistical data analysis

In this study, we evaluated the measurement properties of the BBQ and WHO-5 instruments, guided primarily by the COSMIN (COnsensus-based Standards for the selection of health Measurement INstruments) checklist [44]. Specifically, we assessed internal consistency, structural validity, construct validity, and measurement invariance. However, we did not evaluate measurement error or responsiveness.

Various statistical analyses were used for this study. For descriptive statistics, the general distributions of study variables among the healthcare workers were assessed using frequencies and percentages for categorical variables and means and standard deviation for continuous variables. The internal consistencies of the BBQ and WHO-5 scales were evaluated using Cronbach’s alpha and McDonald’s omega [45]. This study used Cronbach’s Alpha and McDonald’s Omega because Cronbach’s Alpha assumes tau-equivalence, which implies that all items have equal weight in measuring the underlying construct. McDonald’s Omega addresses the potential underestimation bias of Alpha when the assumption of tau-equivalence is not met [46]. A threshold of 0.7 was used to indicate good internal consistency [47]**.** Additionally, ‘alpha if item deleted’ values were computed to evaluate each item’s importance to the overall scale. If the overall Cronbach’s alpha increased by more than 0.02 after deleting an item, it was considered that the item might negatively impact the scale’s internal consistency.

The Kaiser-Meyer-Olkin (KMO) measure and Bartlett’s test of sphericity were used to confirm whether the dataset was sufficient for factor analysis. Having KMO estimates above 0.7 and a significant Bartlett’s test indicated, were thresholds for sample adequacy [47]**.** Confirmatory factor analysis (CFA) was employed to evaluate the scale’s construct validity [48]**.** CFA model fitness was assessed using the Root Mean Square Error of Approximation (RMSEA), Standardized Root Mean Square Residual (SRMR), Comparative Fit Index (CFI), and Tucker-Lewis Index (TLI). The CFA models were considered adequate if the RMSEA < 0.06, the SRMR < 0.08, the CFI > 0.90, and the TLI > 0.90 [49]. For each CFA model, all items were treated as ordered categorically by fitting the models using weighted least square means and variance (WLSMV, theta parameterization). Factor loadings greater than 0.30 indicated a strong relationship between the items and factors [50,51].

Furthermore, measurement invariance of the BBQ and WHO-5 across Swahili and English versions were evaluated after appending the nurses, midwives and CHVs datasets. A sequence of invariances was considered, configural invariance versus metric invariance and scalar invariance versus metric invariance, where ∆CFI < 0.01 indicated the scales were generalizable across the two languages [52].

Recent studies have reported that QoL and well-being are negatively related to anxiety, perceived stress, and depression [53]. Therefore, divergent validity was evaluated by correlating the BBQ and WHO-5 scores with the total scores of the GAD-7, PSS-10, and PHQ-9. The Spearman correlation was used to evaluate the divergent validity.

Linear regression was used to assess the socio-demographic, psychological factors, and health system measures associated with QoL and well-being of nurses/midwives and community health volunteers. Both univariate and multivariate linear regressions were fitted. The backward stepwise procedure was employed, and all variables with a p-value < 0.2 were included in the multivariate analysis. The model assumptions were evaluated using the diagnostics plots and formal statistics. The scatter plots were used to assess the linearity assumption, the Shapiro-Wilk normality test was used to test check for the homoscedasticity of the error terms, the Bonferroni test was used to check for the outliers, and the variance inflation factor (VIF) was used to check for multicollinearity where VIF < 10 indicated there is no multicollinearity [44]. All statistical analyses were performed at the 5% level of significance and analyzed using R version 4.1.2, open-source software for statistical computing and graphics (R Core Team, 2016).

## Results

The study consisted of 2027 CHVs and 1907 nurses/midwives. The socio-demographic characteristics of the study participants are summarized in **Table 1**. For CHVs, the average age was 43.93 (SD = 11.05) years. More than half of CHVs were females (52.8%), married (79.0%), lived with their partners (76.2%), received psychosocial support from religion (53.9%), and had a work experience of less than ten years (68.5%). During COVID-19, 44.6% of the CHVs reduced their working hours, 20.7% were directly exposed to COVID-19, and 1.9% tested positive for COVID-19. The characteristics of the nurses/midwives group results showed that the average age was 34.2 (SD = 10.28), and the majority were females (59.9%). Most of the nurses/midwives had a diploma in nursing/midwifery (72.4%), were married (56.7%), received psychosocial support from religion (67.6%), had a working experience of less than ten years (72.0%), worked in a public health facility (53.9%), and worked between 8–11 hours a day (64.9%). During COVID-19, 36.1% of nurses/midwives increased their working hours, 68.7% were directly exposed to COVID-19, and 15.4% tested positive for COVID-19.

**Table 1:** Socio-demographic characteristics of community health volunteers and nurses/midwives.

	Community Health Volunteers	Nurses/Midwives
	**n (%)**	**n (%)**
Over-all	2027	1907
**Sex**		
Female	1070 (52.8)	1143 (59.9)
Male	957 (47.2)	764 (40.1)
**Age** *means (SD)*	43.93 ± 11.05	34.12 ± 10.28
**Education level**		
Secondary school	896 (44.2)	–
Nursing/midwifery (certificate)	3 (0.1)	73 (3.8)
Nursing/midwifery (Diploma)	5 (0.2)	1381 (72.4)
BSc nursing	0 (0)	346 (18.1)
MSc nursing	0 (0)	35 (1.8)
Other	1123 (55.4)	72 (3.8)
**Marital status**		
Single	213 (10.5)	774 (40.6)
Married	1601 (79.0)	1082 (56.7)
Divorced/Separated	62 (3.1)	28 (1.5)
Widowed/Widower	151 (7.4)	23 (1.2)
**Living with Partner**		
Yes	1544 (76.2)	902 (47.3)
No	57 (2.8)	180 (9.4)
*Missing*	426 (21.0)	825 (43.3)
**Psychosocial support from religious institutions**		
Yes	1092 (53.9)	1290 (67.6)
No	775 (38.2)	524 (27.5)
*Missing*	160 (7.9)	93 (4.9)
**Work duration**		
< 10 years	1388 (68.5)	1373 (72.0)
11 – 20 years	557 (27.5)	263 (13.8)
21 - 30 years	53 (2.6)	175 (9.2)
31- 40 years	7 (0.3)	63 (3.3)
>40 years	5 (0.2)	5 (0.3)
*Missing*	17 (0.8)	28 (1.5)
**Health facility management**		
Public	–	1028 (53.9)
Private	–	689 (36.1)
Faith based organization	–	145 (7.6)
Others	–	45 (2.4)
**Working hours/day**		
< 8	1883 (92.9)	195 (10.2)
8 – 11	112 (5.5)	1237 (64.9)
12 -16	23 (1.1)	453 (23.8)
> 16	9 (0.4)	22 (1.2)
**Change in working hours during COVID-19**		
Decreased	904 (44.6)	264 (13.8)
Increased	840 (41.4)	689 (36.1)
Remained the same	283 (14.0)	954 (50.0)
**Health insurance**		
Yes	785 (38.7)	1743 (91.4)
No	1242 (61.3)	164 (8.6)
**Directly exposed to COVID-19**		
Yes	420 (20.7)	1311 (68.7)
No	1607 (79.3)	596 (31.3)
**Tested positive for COVID-19**		
Yes	39 (1.9)	293 (15.4)
No	1988 (98.1)	1614 (84.6)

### Internal consistency

**Table 2** shows the results for Cronbach’s alpha and McDonald’s omega of the BBQ and WHO-5. The alpha values ranged from 0.73 to 0.80, whereas omega ranged from 0.72 to 0.80. The scales portrayed good internal consistency among CHVs and nurses/midwives.

**Table 2:** Reliability Coefficients for Brunnsviken Brief Quality of Life scale and WHO-5 Well-being scale.

	Cronbach’s alpha (95% CI)	McDonald’s omega (95% CI)
**Community Health Volunteers**		
Brunnsviken Brief Quality of life scale (BBQ)	0.80 (0.79 – 0.81)	0.80 (0.78 – 0.81)
WHO-5 Well-being scale	0.77 (0.76 – 0.79)	0.77 (0.75 – 0.79)
**Nurses/midwives**		
Brunnsviken Brief Quality of life scale (BBQ)	0.73 (0.71 – 0.74)	0.72 (0.70 – 0.74)
WHO-5 Well-being scale	0.79 (0.77 – 0.80)	0.79 (0.78 – 0.81)

***Note:*** *alpha or omega >0.70 implies that the scale has a good internal consistency.*

### Divergent validity

**Table 3** provides the Spearman correlation of BBQ and WHO-5 with GAD-7, PSS-10, and PHQ-9 across CHVs and nurses/midwives. The results for nurses/midwives showed that both BBQ and WHO-5 were significantly negatively correlated with GAD-7, PSS-10, and PHQ-9, demonstrating divergent validity. Similarly, among the CHVs, WHO-5 was found to be significantly negatively correlated with GAD-7, PSS-10, and PHQ-9, also indicating divergent validity.

**Table 3:** Correlation of BBQ and WHO-5 with the GAD-7, PSS-10, and PHQ-9.

	Community Health Volunteers		Nurses/midwives	
	BBQ	WHO-5	BBQ	WHO-5
Divergent				
GAD-7	-0.02	-0.22*	-0.21*	-0.43*
PSS-10	-0.13	-0.25*	-0.23*	-0.42*
PHQ-9	-0.03	-0.23*	-0.22*	-0.43*

### Construct validity

First, we evaluated the factor structure of the BBQ and the WHO-5 questionnaires using the Kaiser–Meyer–Olkin (KMO) and Bartlett’s test of sphericity. The KMO estimates were above 0.80, while Bartlett’s test was significant (p < 0.001) for both BBQ and WHO-5, indicating that the tools had adequate factorial structures for CHVs and nurses/midwives, respectively.

**S1 Table** presents both measures’ score distributions, alpha if item is deleted, and factor loadings of scale items across study groups. All factor loadings for BBQ and WHO-5 items were significant and exceeded the cut-off threshold of 0.35. **Table 4** represents the results of the Confirmatory Factor Analysis (CFA). The CFA results revealed both BBQ and WHO-5 had good construct validity. The chi-square results were significant for all CFA models, portraying the models had an inadequate fit, but this is unreliable because the sample size usually influences the chi-square. We therefore used other fit indexes to evaluate the measure. The RMSEA ranged from 0.032 – 0.061, SRMR ranged from 0.023 – 0.064, and both TLI and CFI values were above.09. Such ranges indicated the two scales had acceptable CFA model fits. The factor loadings were all significant and within the acceptable range (>.30).

**Table 4:** Goodness of fit indices for the unidimensional structure of BBQ and WHO-5.

Fit Indices	Chi-square statistic	RMSEA	SRMR	TLI	CFI
**Community Health Volunteers**					
Brunnsviken Brief Quality of life scale (BBQ)	χ2 (54, n = 2027) = 255.23, p < 0.001	0.043 (0.038 - 0.048)	0.054	0.955	0.963
WHO-5 Item Well-being Index	χ2 (5, n = 2027) = 15.53, p = 0.008	0.032 (0.015 - 0.051)	0.023	0.993	0.997
**Nurses/midwives**					
Brunnsviken Brief Quality of life scale (BBQ)	χ^2^ (54, n = 1907) = 436.87, p < 0.001	0.061 (0.056 - 0.066)	0.064	0.901	0.919
WHO-5 Item Well-being Index	χ2 (10, n = 1907) = 25.57, p < 0.001	0.046 (0.030 – 0.065)	0.031	0.988	0.994

### Measurement invariance across languages used for BBQ and WHO-5 scales

The construct validity was further evaluated using measurement invariance to ensure a unidimensional factor of the two scales across Swahili and English versions (note; total samples of CHVs and nurses/midwives were combined to examine the measurement invariance). The results revealed the unidimensional BBQ and WHO-5 across the languages (**Table 5**).

**Table 5:** Goodness of fit indices for measurement invariance of the BBQ and the WHO-5.

	Fit Indices	Brunnsviken Brief Quality of life scale (BBQ)	WHO-5 Item Well-being Index
***Kiswahili vs English***			
Configural invariance	Chi-square statistic	χ2 (162, n = 3930) = 780.519, p < 0.001	χ2 (15, n = 3930) = 43.040, p < 0.001
	RMSEA (90% CI)	0.054 (0.050 – 0.058)	0.038 (0.025 – 0.051)
	SRMR	0.058	0.024
	TLI	0.926	0.991
	CFI	0.939	0.996
	∆CFI	–	
Metric invariance	Chi-square statistic	χ2 (184, n = 3930) = 847.010, p < 0.001	χ2 (23, n = 3930) = 59.580, p = 0.002
	RMSEA (90% CI)	0.052 (0.049 – 0.056)	0.028 (0.016 – 0.040)
	SRMR	0.060	0.025
	TLI	0.930	0.995
	CFI	0.935	0.996
	∆CFI	0.004	<0.01
Scalar invariance	Chi-square statistic	χ2 (206, n = 3930) = 1370.257, p < 0.001	χ2 (31, n = 3930) = 46.933, p = 0.002
	RMSEA (90% CI)	0.066 (0.062 – 0.069)	0.027 (0.016 – 0.037)
	SRMR	0.071	0.028
	TLI	0.890	0.996
	CFI	0.886	0.996
	∆CFI	0.049	<0.01

The overall measurement invariance of the model by estimating all parameters freely across the languages (configural invariance) had adequate fit indices (**Table 5**). After constraining the factor loadings to be equal across the groups (metric invariance), the models showed good fit indices (**Table 5**). The change of CFI between the configural invariance and metric invariance for the two scales was < 0.01, which indicated full metric invariance. Both factor loadings and intercepts were then constrained (scalar invariance) to be equal across the groups, resulting in good fit indices (**Table 5**). The ∆CFI between metric invariance and scalar invariance for the WHO-5 was < 0.01, which portrayed full scalar invariance, whereas for the BBQ was 0.049, indicating partial scalar invariance.

### Factors associated with quality of life and well-being among community health volunteers

**S2 Table** presents the results of univariate analysis. **Table 6** provides the beta coefficient estimates, their 95% confidence interval (CI) and P-values from the multiple linear regression model that evaluates the sociodemographic characteristics, COVID-19-related factors, psychosocial outcomes, and health system domains predicting the quality of life and well-being among the CHVs. The results show that the females had lower scores on well-being than males (ß = -0.86, p = .001). On average, the well-being scores among the CHVs decreased by 0.23 units for every unit increase in burnout (ß = -0.23, p < .001), 0.03 for every unit increase in stigma (ß = -0.03, p = .017), and 0.26 for every unit increase in post-traumatic stress disorder (ß = -0.26, p < .001). Contrary, the well-being scores among CHVs increased by 0.08 for a unit increase in work engagement (ß = 0.08, p < .0001), 0.07 for a unit increase in social support (ß = 0.07, p < .001), and 0.09 on a unit increase in attitude towards seeking psychosocial support (ß = 0.09, p = .004). Similarly, for every unit increase in an individual capacity and motivation score, job factors, working environment, management and leadership, structural support, and perceptions of health system capacity increased the well-being scores among CHVs.

**Table 6:** Multiple linear regression estimates for predictors of QoL and well-being among nurses/midwives and CHVs.

	Community Health Volunteers				Nurses/midwives			
	Brunnsviken Brief Quality of life		WHO-5 Well-being		Brunnsviken Brief Quality of life		WHO-5 Well-being	
	ß (95% CI)	P value	ß (95% CI)	P value	ß (95% CI)	P value	ß (95% CI)	P value
**Socio demographic characteristics**								
**Sex**								
Male	*Reference*							
Female	-1.00 (-2.08, 0.09)	0.073	**-0.86 (-1.35, -0.36)**	**0.001**			**-0.61 (-1.03, -0.18)**	**0.005**
**Age**	**-0.05 (-0.10, 0.00)**	**0.032**			-0.05 (-0.12, 0.02)	0.131		
**Education level**								
BSc nursing	*Reference*							
Nursing/midwifery (certificate)	–	–	–	–	-2.62 (-5.92,0.68)	0.120	**1.46 (0.29,2.63)**	**0.015**
Nursing/midwifery (Diploma)	–	–	–	–	-1.5 (-3.04,0.04)	0.056	0.27 (-0.27,0.82)	0.326
MSc nursing	–	–	–	–	1.34 (-3.12,5.8)	0.556	-0.11 (-1.7,1.49)	0.897
Other	–	–	–	–	-1.2 (-4.48,2.08)	0.474	-0.94 (-2.11,0.23)	0.114
**Marital status**								
Single	*Reference*							
Married					**-1.34 (-2.64, -0.03)**	**0.044**		
Divorced/Separated					1.87 (-3.09, 6.83)	0.461		
Widowed/Widower					-4.42 (-9.93, 1.10)	0.117		
**Psychosocial support from religion**								
Yes	*Reference*							
No					**-1.33 (-2.62, -0.04)**	**0.043**		
**Health Facility**								
Public	*Reference*							
Private	**–**	**–**	–	–	**-1.40 (-2.75, -0.05)**	**0.042**		
Faith based organization	**–**	**–**	–	–	1.01 (-1.28, 3.30)	0.389		
Others	**–**	**–**	–	–	-0.40 (-4.26, 3.45)	0.837		
**Work duration**								
< 10 years	*Reference*							
11 – 20 years								
21 - 30 years	1.52 (-0.84,3.88)	0.208						
31- 40 years								
>40 years								
**Working hours/day**								
< 8	*Reference*							
8 – 11	-0.85 (-3.19, 1.50)	0.480					**-0.89 (-1.59, -0.19)**	**0.012**
12 -16	-1.82 (-6.80, 3.17)	0.475					**-1.08 (-1.86, -0.31)**	**0.006**
> 16	-7.57 (-15.69, 0.55)	0.068					**-2.75 (-4.78, -0.72)**	**0.008**
**Change in working hours during COVID-19**								
Decreased	*Reference*							
Increased	0.82 (-0.35, 1.98)	0.169						
Remained the same	-0.37 (-2.02, 1.29)	0.664						
**Directly exposed to COVID-19**								
Yes	*Reference*							
No	**-1.62 (-3.00, -0.24)**	**0.021**			-0.87 (-2.19, 0.44)	0.193	**0.72 (0.26, 1.18)**	**0.002**
**Tested positive for COVID-19**								
Yes	*Reference*							
No	2.96 (-1.09, 7.00)	0.152			-1.36 (-3.03, 0.31)	0.112		
**Psychological health**								
Resilience					**0.29 (0.12, 0.47)**	**0.001**	**0.18 (0.12, 0.25)**	**< 0.001**
Burnout	-0.67 (-0.83, -0.51)	**< 0.001**	**-0.23 (-0.31, -0.15)**	**< 0.001**	-0.15 (-0.30, 0.01)	0.058	**-0.24 (-0.3, -0.19)**	**< 0.001**
Stigmatization Survey			**-0.03 (-0.06, -0.01)**	**0.017**	0.04 (-0.02, 0.10)	0.178		
Work Engagement			0.08 (0.06, 0.11)	**< 0.001**	**0.07 (0.01, 0.14)**	**0.034**	0.05 (0.03,0.08)	**< 0.001**
Social Support	0.25 (0.19, 0.32)	**< 0.001**	0.07 (0.04, 0.10)	**< 0.001**	0.44 (0.36, 0.51)	**< 0.001**	0.06 (0.04,0.09)	**< 0.001**
Attitude towards seeking psychosocial support	**0.44 (0.31, 0.57)**	**< 0.001**	0.09 (0.03, 0.14)	0.004	**0.17 (0.01, 0.34)**	**0.039**		
Post-traumatic stress disorder			-0.26 (-0.40, -0.12)	**< 0.001**			**-0.33 (-0.44, -0.21)**	**< 0.001**
**Health system domain**								
Individual capacity and motivation	**0.97 (0.77,1.17)**	**< 0.001**	**0.08 (-0.01,0.18)**	**0.090**	**0.56 (0.36, 0.76)**	**< 0.001**	**0.09 (0.02,0.17)**	**0.010**
Job factors			**0.2 (0.11, 0.29)**	**< 0.001**	0.15 (-0.05, 0.36)	0.138	**0.15 (0.08, 0.22)**	**< 0.001**
Work environment			**0.07 (0.02,0.11)**	**0.004**	**0.11 (0.03, 0.20)**	**0.009**		
Management and leadership	**0.80 (0.47,1.13)**	**< 0.001**	**-0.18 (-0.33, -0.03)**	**0.020**			**0.06 (0.01, 0.10)**	**0.010**
Structural support	0.08 (-0.05,0.21)	0.215	**0.03 (-0.03,0.09)**	**0.380**	0.02 (-0.10, 0.15)	0.738		
Perceptions of health system capacity	**0.32 (0.07,0.57)**	**0.013**	**0.18 (0.07,0.3)**	**0.002**	**0.21 (0.01, 0.42)**	**0.043**		

The quality-of-life scores of the CHVs decreased by 0.05 for every year of age increase (ß = -0.05, p = .032). CHVs who were directly exposed to COVID-19 had a lower quality of life score than those who were not (ß = -1.62, p = .021). The findings revealed an improvement in quality of life for a unit increase in social support score (ß = 0.25, p < .001) and attitude toward seeking psychosocial support (ß = 0.44, p < .001). In terms of health system domains, a unit increase in an individual capacity and motivation scores increased QoL scores by 0.97 (ß = 0.97, p < .001), a unit increase in management and leadership scores increased quality-of-life scores by 0.80 (ß = 0.80, p < .001), and a unit increase in perceptions of health system capacity increased quality-of-life scores by 0.32 (ß = 0.32, p = .013).

### Factor associated with quality of life and well-being among nurse/midwives

**S2 Table** presents the results of univariate analysis**.** The sociodemographic characteristics, COVID-19-related questions, psychosocial outcomes, and health system domain predicting the quality of life and well-being on multivariate analysis among nurses/midwives are shown in **Table 6**. The result indicates that the nurse/midwives with a nursing certificate had higher scores on quality of life than those with a BSc in nursing (ß = 1.46, p = .015). Females had a lower quality of life score than the males (ß = -0.61, p = .005). The nurses/midwives who worked more than eight hours per day had a lower well-being score than those who worked less than eight hours per day (**Table 6**). Nurses/midwives not exposed to COVID-19 had a 0.72-unit higher well-being score than those exposed (ß = 0.72, p = .002). In terms of psychosocial outcomes, a unit increase in resilience increased the score on well-being by 0.18 (ß = 0.18, p < .001), a unit increase in work engagement increased the well-being score by 0.05 (ß = 0.05, p < .001), and a unit increase in social support increased the well-being score by 0.06 (ß = 0.06, p < .001). A unit increase in burnout (ß = -0.24, p < .001) and post-traumatic stress disorder (ß = -0.33, p = .001) were linked to a 0.24 and 0.33 unit decrease in well-being, respectively. The results show that a unit increase in an individual capacity and motivation increased well-being scores by 0.09 (ß = 0.09, p = .010), a unit increase in job factors increased well-being scores by 0.15 (ß = 0.15, p < .001), and a unit increase in management and leadership increased well-being scores by 0.06 (ß = 0.06, p = .010).

Being married versus single had a lower quality of life score among nurses/midwives (ß = -1.34, p = .044). The QoL of nurses/midwives who received psychosocial support from religion was 1.33 units lower than those who did not receive support (ß = -1.33, p = .004). Furthermore, nurses/midwives who worked in private health facilities had 1.40 lower QoL scores than those who worked in public facilities (ß = -1.40, p = .042). The average QoL score increased by 0.29 units for every unit increase in resilience in the category of psychosocial outcomes (ß = 0.29, p = .001). Consequently, every unit increase in individual capacity and motivation, work environment, and perceptions of health system capacity improved nurses’ and midwives’ QoL (**Table 6**).

## Discussion

### Summary of findings

Quality of life and well-being among healthcare workers is critical because it directly impacts positive outcomes for both healthcare providers and patients [54]. Identification of valid and reliable tools for screening this population’s quality of life and well-being is important in understanding and improving their welfare. This study aimed to assess the psychometric properties of the Brunnsviken Brief Quality of Life Scale (BBQ) and WHO-5 well-being tools, and the factors associated with quality of life and well-being among nurses/midwives and CHVs in a national-wide survey in Kenya. The Cronbach’s alpha and MacDonald’s omega results indicated that BBQ and WHO-5 had good internal consistency for nurses/midwives and CHVs, respectively. Furthermore, CFA results showed that the BBQ and WHO-5 had a unidimensional factor, and measurement variance results revealed that the BBQ and WHO-5 had an invariant one factor across the Swahili and English versions. The multiple linear regression model results showed that being male, having no burnout, being resilient, having good work engagement, receiving social support, and having good health systems support all improved the quality of life and well-being of CHVs. Also, the multiple linear models revealed that receiving psychosocial support from religion, resilience, work engagement, and good health systems were all positively associated with nurses’/midwives’ quality of life and well-being. Burnout, stigma, and PTSD reduced nurses’ and midwives’ quality of life and well-being.

### Internal consistency

Our findings indicated that both the BBQ and WHO-5 well-being index tools demonstrated good internal consistency (Cronbach’s alpha and Macdonald’s omega > 0.7) for nurses/midwives and CHVs. These findings suggest that the items in these tools were correlated and effectively measured quality of life and well-being, respectively. Therefore, the BBQ and WHO-5 well-being index can be considered reliable for assessing healthcare workers’ quality of life and well-being. These findings align with previous research validating the WHO-5 well-being index among nurses in Spain, Chile, and Norway (alpha > 0.8) [55], validating the WHO-5 well-being index among medical educators in Hong Kong [56] and on a different population of adults living with HIV and epilepsy in Kenya [57]. The findings of good internal consistency of the BBQ concur with its reliability in clinical and research settings [24].

### Construct validity

The CFA analysis confirmed the one-factor structure for the BBQ and WHO-5 well-being index. All factor loadings for BBQ and WHO-5 items were significant and exceeded the cut-off threshold of 0.35. These findings indicate a robust and positive association between the items and the underlying constructs of quality of life and well-being, respectively. Additionally, these findings affirm the usability of the BBQ and the WHO-5 as reliable measures of quality of life and well-being among the CHVs and nurses/midwives. The one factor structure of the WHO-5 well-being index finding aligns with the validation of WHO-5 well-being index among nurses across Spain, Chile, and Norway during COVID-19 pandemic where the unidimensional factor structure was confirmed across the three countries [55]. The unidimensionality of WHO-5 is consistent with previous research findings in low-income countries [57,58]. Our findings are consistent with the BBQ’s psychometric evaluation during its development, which involved two distinct populations: health undergraduate students and individuals seeking help for social anxiety disorder [24].

The measurement invariance analysis demonstrated that the BBQ and WHO-5 were invariant across both the Swahili and English versions. These findings suggest that the Swahili and English versions of the BBQ and WHO-5 are suitable for evaluating the quality of life and well-being of healthcare workers. Previous studies have reported the generalizability of WHO-5 across different groups [58,59]. Noteworthy, based on our knowledge, no study had reported the measurement invariance of BBQ across groups. However, full scalar invariance was not achieved for the BBQ indicating that not all item function equivalently across subgroups. This may limit the validity of direct comparisons of observed scores, potentially due to response biases or item variations among participants using different versions. However, this does not undermine the generalizability of the BBQ Swahili and English versions, and further research is necessary to explore item wording or formulation [60]. Additionally, we suggest that future studies conduct differential item functioning (DIF) analyses to ensure that the BBQ measures the construct equivalently across the Swahili and English versions.

### Divergent validity

The results revealed distinct patterns of negative correlations between the WHO-5 and BBQ with PSS-10, GAD-7, and PHQ-9 among nurses/midwives, while WHO-5 with PSS-10, GAD-7, and PHQ-9 among community health volunteers. The negative correlation results suggest that higher levels of well-being, as measured by WHO-5, are associated with lower levels of anxiety, stress, and depression among CHWs. In contrast, both the high quality of life and well-being, as measured by BBQ and WHO-5, lower anxiety, stress, and depression levels, indicating divergent validity of these measures among nurses/midwives. Our results are consistent with other studies among healthcare workers conducted during COVID-19 indicating that depression, stress and anxiety had a negative impact on the quality of life and well-being [61–64].

### Key predictors of well-being and quality of life

One key finding reveals that both female CHVs and nurses/midwives experience poorer well-being scores compared to males. This disparity may be attributed to the fact that a significant number of female CHVs and nurses/midwives were married, leading to additional caregiving responsibilities. We speculate that, balancing these responsibilities with the demands of their profession could have a negative impact on their overall well-being as has earlier been reported [65]. The results showed that married nurses and midwives had poorer quality of life scores compared to those who were single. This gap could be attributable to the increased domestic and caregiving tasks that married individuals, particularly women, frequently shoulder, such as childcare, and household administration. These additional expectations may worsen work-related stress hence leading to lower perceived quality of life [66]. The results also revealed that the higher level of burnout negatively impacted the well-being of CHVs and nurses/midwives. This may be due to the increase of workload, stressors and emotional strains experienced by healthcare workers during COVID-19. Our findings are consistent with previous research, highlighting the negative impact of burnout on well-being among healthcare workers in the context of COVID-19 [12,67,68]. Addressing the well-being and quality of life of frontline healthcare workers is critical because earlier studies have reported a correlation between the well-being of healthcare workers and patient welfare [15,61,69].

Another key finding showed that more work engagement among CHVs and nurses/midwives increased their well-being. Bakker et al. provide the Job Demands-Resources (JD-r) model and describes work engagement as a positive psychological state characterized by a high level of vigor, dedication and absorption [70]. This state of engagement may have enhanced the well-being of healthcare workers in this context. These results are consistent with earlier studies from various parts of the globe that indicate that levels of work engagement are associated with different measures of well-being and psychosocial functioning [71–73].

Also, the results showed that high social support increased the well-being for both CHVs and nurses/midwives. Our findings align with the previous findings indicating that the social support particularly from friends and well-being among healthcare workers increased the levels of well-being during COVID-19 [74,75]. For instance, a study by Zhang and colleagues (2022) investigated the psychological distress experienced by HCWs at a tertiary hospital during the early stage of COVID-19 and evaluated the moderating effects of resilience and social support on the relationship between stress and psychological distress. They reported that approximately 1 in every 3 of their participants experienced psychological distress. However, high levels of social support and resilience appear to protect health care workers against stress.

There is ample empirical evidence to indicate that the work environment can have either protective or adverse effects on mental health functioning [76,77]. Our results are consistent with these earlier reports that the health system factors such as individual capacity and motivation, job factors, and the work environment had a positive influence on the well-being among healthcare workers. These set of results imply that it is important to develop intervention programs that address both individual and job-related factors to ensure a positive impact on heath care workers quality of life and well-being.

Our results revealed that receiving psychosocial support from religion was negatively associated with QoL among nurses and midwives. This finding is contrary to what has been reported in the literature, where religious and spiritual support are often associated with improved psychological well-being and coping, particularly in stressful professions such as healthcare [78]. One possible explanation is that healthcare workers who are already experiencing lower QoL may be more likely to seek or rely on religious psychosocial support as a coping mechanism, rather than religious support directly causing lower QoL. Another possible explanation is that religious messages may emphasize endurance and acceptance without addressing systemic workplace stressors, which could limit the effectiveness of religious support in improving QoL. Future studies should explore the nature, quality, and contextual relevance of religious psychosocial support to better understand this complex relationship.

## Strengths and limitations

Our study has strengths and limitations worth highlighting. A key strength of this study was a large nationally representative sample of a diverse range of frontline health worker cadres. Secondly, the study had a distinctive focus on QoL among frontline healthcare workers and to the best of our knowledge is among the first or few in sub-Saharan Africa focused on healthcare workers. Another key strength of this study is that validation was conducted according to key COSMIN guidelines, assessing internal consistency, structural validity, construct validity, and measurement invariance. Regarding limitations, we did not evaluate for temporal stability or test re-test reliability, content validity and measurement error. Further work looking at retest values and predictive validity are warranted. Furthermore, future longitudinal studies could employ the Longitudinal Structural Equation Modelling (LSEM) approaches, such as longitudinal measurement invariance testing and latent growth modelling, to evaluate how the psychometric properties and construct validity of the BBQ and WHO-5 may change over time. Additionally, we only looked at the English and Kiswahili versions, yet Kenya is a multi-lingual country. Future research could explore the measurement invariance of the BBQ and WHO-5 in other cultural or linguistic contexts to further establish their cross-cultural validity. In addition, investigating potential sources of non-invariance, such as cultural differences in response styles, could contribute to refining the measurement instruments.

## Conclusion

The BBQ and WHO-5 scales are reliable and valid tools that can be used to respectively evaluate the quality of life and well-being of healthcare workers in Kenya. Multiple factors influence the well-being and quality of life of health care workers in Kenya. Intervention programs that address both individual and job-related factors can positively impact the quality of life and well-being of healthcare workers.

## Supporting information

S1 Table Score distribution, alpha if item is deleted, and factor loadings of scale items across study groups. (DOCX)

S2 Table Univariate linear regression estimates indicating the association between the total scores of Brunnsviken Brief Quality of life scale and WHO-5 Well-being scale with predictors. (DOCX)

## References

[1] CDC. Health-Related Quality of Life (HRQOL). 2018 13/01/2023. Report No.

[2] The World Health Organization Quality of Life Assessment (WHOQOL): development and general psychometric properties. Soc Sci Med. 1998;46(12):1569–85. doi: 10.1016/s0277-9536(98)00009-4 9672396

[3] Stefanatou P, Xenaki L-A, Karagiorgas I, Ntigrintaki A-A, Giannouli E, Malogiannis IA, et al. Fear of COVID-19 Impact on Professional Quality of Life among Mental Health Workers. Int J Environ Res Public Health. 2022;19(16):9949. doi: 10.3390/ijerph19169949 36011583 PMC9408175

[4] Katschnig H. Quality of life in mental disorders: challenges for research and clinical practice. World Psychiatry. 2006;5(3):139–45. 17139340 PMC1636133

[5] Davis T. What Is Well-Being? Definition, Types, and Well-Being Skills 2019. Available from: https://www.psychologytoday.com/us/blog/click-here-happiness/201901/what-is-well-being-definition-types-and-well-being-skills

[6] Dodge R, Daly A, Huyton J, Sanders L. The challenge of defining wellbeing. Intnl J Wellbeing. 2012;2(3):222–35. doi: 10.5502/ijw.v2i3.4

[7] Sfeatcu R, Cernuşcă-Miţariu M, Ionescu C, Roman M, Cernuşcă-Miţariu S, Coldea L. The concept of wellbeing in relation to health and quality of life. European Journal of Science and Theology. 2014;10(4):123–8.

[8] Upton D, Upton P. Psychology of wounds and wound care in clinical practice: Springer; 2015.

[9] Diener E, Oishi S, Tay L. Advances in subjective well-being research. Nat Hum Behav. 2018;2(4):253–60. doi: 10.1038/s41562-018-0307-6 30936533

[10] Ryan RM, Deci EL. Self-determination theory: Basic psychological needs in motivation, development, and wellness: Guilford Publications; 2017.

[11] Asante JO, Li MJ, Liao J, Huang YX, Hao YT. The relationship between psychosocial risk factors, burnout and quality of life among primary healthcare workers in rural Guangdong province: a cross-sectional study. BMC Health Serv Res. 2019;19(1):447. doi: 10.1186/s12913-019-4278-8 31269949 PMC6610857

[12] Søvold LE, Naslund JA, Kousoulis AA, Saxena S, Qoronfleh MW, Grobler C, et al. Prioritizing the Mental Health and Well-Being of Healthcare Workers: An Urgent Global Public Health Priority. Front Public Health. 2021;9:679397. doi: 10.3389/fpubh.2021.679397 34026720 PMC8137852

[13] Silver SR, Li J, Marsh SM, Carbone EG. Prepandemic Mental Health and Well-being: Differences Within the Health Care Workforce and the Need for Targeted Resources. J Occup Environ Med. 2022;64(12):1025–35. doi: 10.1097/JOM.0000000000002630 36472564 PMC9722331

[14] Aslan H, Erci B, Pekince H. Relationship Between Compassion Fatigue in Nurses, and Work-Related Stress and the Meaning of Life. J Relig Health. 2022;61(3):1848–60. doi: 10.1007/s10943-020-01142-0 33386572 PMC7775832

[15] Babapour A-R, Gahassab-Mozaffari N, Fathnezhad-Kazemi A. Nurses’ job stress and its impact on quality of life and caring behaviors: a cross-sectional study. BMC Nurs. 2022;21(1):75. doi: 10.1186/s12912-022-00852-y 35361204 PMC8968092

[16] Ruiz-Fernández MD, Ortega-Galán ÁM, Fernández-Sola C, Hernández-Padilla JM, Granero-Molina J, Ramos-Pichardo JD. Occupational Factors Associated with Health-Related Quality of Life in Nursing Professionals: A Multi-Centre Study. Int J Environ Res Public Health. 2020;17(3):982. doi: 10.3390/ijerph17030982 32033257 PMC7038014

[17] Deng D, Naslund JA. Psychological impact of COVID-19 pandemic on frontline health workers in low-and middle-income countries. Harvard public health review (Cambridge, Mass). 2020;28. PMC7785092 33409499

[18] Stojanov J, Malobabic M, Stanojevic G, Stevic M, Milosevic V, Stojanov A. Quality of sleep and health-related quality of life among health care professionals treating patients with coronavirus disease-19. Int J Soc Psychiatry. 2021;67(2):175–81. doi: 10.1177/0020764020942800 32674637 PMC7369398

[19] Philip J, Cherian V. Factors Affecting the Psychological Well-being of Health Care Workers During an Epidemic: A Thematic Review. Indian J Psychol Med. 2020;42(4):323–33. doi: 10.1177/0253717620934095 33402793 PMC7746908

[20] Jones S, White S, Ormrod J, Sam B, Bull F, Pieh S, et al. Work-based risk factors and quality of life in health care workers providing maternal and newborn care during the Sierra Leone Ebola epidemic: findings using the WHOQOL-BREF and HSE Management Standards Tool. BMJ Open. 2020;10(11):e032929. doi: 10.1136/bmjopen-2019-032929 PMC7668354 33191248

[21] Odole AC, Ogunlana MO, Odunaiya NA, Oyewole OO, Mbada CE, Onyeso OK, et al. Influence of well-being and quality of work-life on quality of care among healthcare professionals in southwest, Nigeria. Sci Rep. 2023;13(1):7830. doi: 10.1038/s41598-022-25057-w 37188741 PMC10185691

[22] Draper H, Sorell T, Ives J, Damery S, Greenfield S, Parry J, et al. Non-Professional Healthcare Workers and Ethical Obligations to Work during Pandemic Influenza. Public Health Ethics. 2009;3(1):23–34. doi: 10.1093/phe/php021

[23] Pequeno NPF, Cabral NL de A, Marchioni DM, Lima SCVC, Lyra C de O. Quality of life assessment instruments for adults: a systematic review of population-based studies. Health Qual Life Outcomes. 2020;18(1):208. doi: 10.1186/s12955-020-01347-7 32605649 PMC7329518

[24] Lindner P, Frykheden O, Forsström D, Andersson E, Ljótsson B, Hedman E, et al. The Brunnsviken Brief Quality of Life Scale (BBQ): Development and Psychometric Evaluation. Cogn Behav Ther. 2016;45(3):182–95. doi: 10.1080/16506073.2016.1143526 26886248 PMC4867878

[25] Pantić M, Rančić N, Đoković D, Mihajlović G. The Serbian version of the Brunnsviken Brief Quality of life scale: Reliability, validity and psychometric features among population of high school student. Vojnosanitetski pregled. 2022;00:37.

[26] Carlbring P. Brunnsviken Brief Quality of life scale (BBQ) 2024 [cited 2024]. Available from: http://www.bbqscale.com 10.1080/16506073.2016.1143526 PMC4867878 26886248

[27] Grande RAN, Berdida DJE, Santos KCP, Pangket P, Cabansag DI. Structural equation modeling of the relationship between nursing students’ quality of life and academic resilience. J Taibah Univ Med Sci. 2021;17(4):667–77. doi: 10.1016/j.jtumed.2021.11.009 35983443 PMC9356355

[28] Klang A, Westerberg B, Humble MB, Bejerot S. The impact of schizotypy on quality of life among adults with autism spectrum disorder. BMC Psychiatry. 2022;22(1):205. doi: 10.1186/s12888-022-03841-2 35305592 PMC8934003

[29] von Lieres JS, Cauvery G. The impact of social media use on young adults’ quality of life during the COVID-19 pandemic in South India. In: 2022.

[30] Bonsignore M, Barkow K, Jessen F, Heun R. Validity of the five-item WHO Well-Being Index (WHO-5) in an elderly population. Eur Arch Psychiatry Clin Neurosci. 2001;251 Suppl 2:II27-31. doi: 10.1007/BF03035123 11824831

[31] Topp CW, Østergaard SD, Søndergaard S, Bech P. The WHO-5 Well-Being Index: a systematic review of the literature. Psychother Psychosom. 2015;84(3):167–76. doi: 10.1159/000376585 25831962

[32] de Wit M, Pouwer F, Gemke RJBJ, Delemarre-van de Waal HA, Snoek FJ. Validation of the WHO-5 Well-Being Index in adolescents with type 1 diabetes. Diabetes Care. 2007;30(8):2003–6. doi: 10.2337/dc07-0447 17475940

[33] Ghazisaeedi M, Mahmoodi H, Arpaci I, Mehrdar S, Barzegari S. Validity, Reliability, and Optimal Cut-off Scores of the WHO-5, PHQ-9, and PHQ-2 to Screen Depression Among University Students in Iran. Int J Ment Health Addict. 2022;20(3):1824–33. doi: 10.1007/s11469-021-00483-5 33495691 PMC7817067

[34] Eser E, Çevik C, Baydur H, Güneş S, Esgin TA, Öztekin ÇS, et al. Reliability and validity of the Turkish version of the WHO-5, in adults and older adults for its use in primary care settings. Prim Health Care Res Dev. 2019;20:e100. doi: 10.1017/S1463423619000343 32800004 PMC6609969

[35] Faruk O, Chowdhury KUA. Assessing well-being of persons with disabilities: Validation of the WHO-5 well-being index. 2021.

[36] Seb-Akahomen OJ, Okogbenin EO, Obagaye OM, Erohubie PO, Aweh BE. The Reliability and Validity of the 5-Item Who Well-Being Index (WHO-5) amongst Doctors and Nurses in Nigeria. West Afr J Med. 2022;39(7):708–13. 35924987

[37] Odero SA, Mwangi P, Odhiambo R, Nzioka BM, Shumba C, Ndirangu-Mugo E. Psychometric evaluation of PHQ-9 and GAD-7 among community health volunteers and nurses/midwives in Kenya following a nation-wide telephonic survey. 2023. 10.3389/fpsyt.2023.1123839 PMC10264862 37324823

[38] Cohen S, Kamarck T, Mermelstein R. A global measure of perceived stress. J Health Soc Behav. 1983;24(4):385–96. doi: 10.2307/2136404 6668417

[39] Maslach C, Jackson SE, Leiter MP. Maslach burnout inventory: Scarecrow Education; 1997.

[40] Park J-S, Lee E-H, Park N-R, Choi YH. Mental Health of Nurses Working at a Government-designated Hospital During a MERS-CoV Outbreak: A Cross-sectional Study. Arch Psychiatr Nurs. 2018;32(1):2–6. doi: 10.1016/j.apnu.2017.09.006 29413067 PMC7127092

[41] Smith BW, Dalen J, Wiggins K, Tooley E, Christopher P, Bernard J. The brief resilience scale: assessing the ability to bounce back. Int J Behav Med. 2008;15(3):194–200. doi: 10.1080/10705500802222972 18696313

[42] Zimet GD, Dahlem NW, Zimet SG, Farley GK. The Multidimensional Scale of Perceived Social Support. Journal of Personality Assessment. 1988;52(1):30–41. doi: 10.1207/s15327752jpa5201_2 2280326

[43] Schaufeli WB, Bakker AB, Salanova M. Utrecht work engagement scale-9. Educational and Psychological Measurement. 2003.

[44] Mokkink LB, Terwee CB, Patrick DL, Alonso J, Stratford PW, Knol DL, et al. The COSMIN checklist for assessing the methodological quality of studies on measurement properties of health status measurement instruments: an international Delphi study. Qual Life Res. 2010;19(4):539–49. doi: 10.1007/s11136-010-9606-8 20169472 PMC2852520

[45] Bacon DR, Sauer PL, Young M. Composite Reliability in Structural Equations Modeling. Educational and Psychological Measurement. 1995;55(3):394–406. doi: 10.1177/0013164495055003003

[46] Dunn TJ, Baguley T, Brunsden V. From alpha to omega: a practical solution to the pervasive problem of internal consistency estimation. Br J Psychol. 2014;105(3):399–412. doi: 10.1111/bjop.12046 24844115

[47] Shrestha N. Factor Analysis as a Tool for Survey Analysis. AJAMS. 2021;9(1):4–11. doi: 10.12691/ajams-9-1-2

[48] Brown TA. Confirmatory factor analysis for applied research: Guilford publications; 2015.

[49] Hu L, Bentler PM. Cutoff criteria for fit indexes in covariance structure analysis: Conventional criteria versus new alternatives. Structural Equation Modeling: A Multidisciplinary Journal. 1999;6(1):1–55. doi: 10.1080/10705519909540118

[50] Fein EC, Gilmour J, Machin T, Hendry L. Statistics for Research Students: An Open Access Resource with Self-Tests and Illustrative Examples. University of Southern Queensland. 2022.

[51] Hu L, Bentler PM. Cutoff criteria for fit indexes in covariance structure analysis: Conventional criteria versus new alternatives. Structural Equation Modeling: A Multidisciplinary Journal. 1999;6(1):1–55. doi: 10.1080/10705519909540118

[52] Cheung GW, Rensvold RB. Evaluating Goodness-of-Fit Indexes for Testing Measurement Invariance. Structural Equation Modeling: A Multidisciplinary Journal. 2002;9(2):233–55. doi: 10.1207/s15328007sem0902_5

[53] Choi H-J, Yang C-M, Lee S-Y, Lee H-J, Jang S-H. Mental Health and Quality of Life for Healthcare Workers in a University Hospital Under COVID-19. Psychiatry Investig. 2022;19(2):85–91. doi: 10.30773/pi.2021.0307 34915610 PMC8898611

[54] Smart D, English A, James J, Wilson M, Daratha KB, Childers B, et al. Compassion fatigue and satisfaction: a cross-sectional survey among US healthcare workers. Nurs Health Sci. 2014;16(1):3–10. doi: 10.1111/nhs.12068 23663318

[55] Lara-Cabrera ML, Betancort M, Muñoz-Rubilar A, Rodríguez-Novo N, Bjerkeset O, De Las Cuevas C. Psychometric Properties of the WHO-5 Well-Being Index among Nurses during the COVID-19 Pandemic: A Cross-Sectional Study in Three Countries. Int J Environ Res Public Health. 2022;19(16):10106. doi: 10.3390/ijerph191610106 36011741 PMC9407690

[56] Chan L, Liu RKW, Lam TP, Chen JY, Tipoe GL, Ganotice FA. Validation of the World Health Organization Well-Being Index (WHO-5) among medical educators in Hong Kong: a confirmatory factor analysis. Med Educ Online. 2022;27(1):2044635. doi: 10.1080/10872981.2022.2044635 35275804 PMC8920356

[57] Chongwo E, Ssewanyana D, Nasambu C, Mwangala PN, Mwangi PM, Nyongesa MK, et al. Validation of a Swahili version of the World Health Organization 5-item well-being index among adults living with HIV and epilepsy in rural coastal Kenya. Glob Health Res Policy. 2018;3:26. doi: 10.1186/s41256-018-0081-z 30214943 PMC6130066

[58] Perera BPR, Jayasuriya R, Caldera A, Wickremasinghe AR. Assessing mental well-being in a Sinhala speaking Sri Lankan population: validation of the WHO-5 well-being index. Health Qual Life Outcomes. 2020;18(1):305. doi: 10.1186/s12955-020-01532-8 32912245 PMC7488505

[59] Krieger T, Zimmermann J, Huffziger S, Ubl B, Diener C, Kuehner C, et al. Measuring depression with a well-being index: further evidence for the validity of the WHO Well-Being Index (WHO-5) as a measure of the severity of depression. J Affect Disord. 2014;156:240–4. doi: 10.1016/j.jad.2013.12.015 24412323

[60] Vandenberg RJ, Lance CE. A Review and Synthesis of the Measurement Invariance Literature: Suggestions, Practices, and Recommendations for Organizational Research. Organizational Research Methods. 2000;3(1):4–70. doi: 10.1177/109442810031002

[61] Gu B, Tan Q, Zhao S. The association between occupational stress and psychosomatic wellbeing among Chinese nurses: A cross-sectional survey. Medicine (Baltimore). 2019;98(22):e15836. doi: 10.1097/MD.0000000000015836 31145327 PMC6708716

[62] Kandula UR, Wake AD. Assessment of quality of life among health professionals during COVID-19. Journal of Multidisciplinary Healthcare. 2021;3571–85. 35002247 10.2147/JMDH.S344055 PMC8722681

[63] Sharma SK, Mudgal SK, Thakur K, Parihar A, Chundawat DS, Joshi J. Anxiety, depression and quality of life (QOL) related to COVID-19 among frontline health care professionals: A multicentric cross-sectional survey. J Family Med Prim Care. 2021;10(3):1383–9. doi: 10.4103/jfmpc.jfmpc_2129_20 34041183 PMC8140249

[64] Suryavanshi N, Kadam A, Dhumal G, Nimkar S, Mave V, Gupta A, et al. Mental health and quality of life among healthcare professionals during the COVID-19 pandemic in India. Brain Behav. 2020;10(11):e01837. doi: 10.1002/brb3.1837 32918403 PMC7667343

[65] Liu S, Yang L, Zhang C, Xu Y, Cai L, Ma S, et al. Gender differences in mental health problems of healthcare workers during the coronavirus disease 2019 outbreak. J Psychiatr Res. 2021;137:393–400. doi: 10.1016/j.jpsychires.2021.03.014 33765451 PMC7962932

[66] Adisa TA, Aiyenitaju O, Adekoya OD. The work–family balance of British working women during the COVID-19 pandemic. JWAM. 2021;13(2):241–60. doi: 10.1108/jwam-07-2020-0036

[67] Xiao Y, Dong D, Zhang H, Chen P, Li X, Tian Z, et al. Burnout and Well-Being Among Medical Professionals in China: A National Cross-Sectional Study. Front Public Health. 2022;9:761706. doi: 10.3389/fpubh.2021.761706 35111713 PMC8801677

[68] Denning M, Goh ET, Tan B, Kanneganti A, Almonte M, Scott A, et al. Determinants of burnout and other aspects of psychological well-being in healthcare workers during the Covid-19 pandemic: A multinational cross-sectional study. PLoS One. 2021;16(4):e0238666. doi: 10.1371/journal.pone.0238666 33861739 PMC8051812

[69] Kirk K. Time for a rebalance: psychological and emotional well-being in the healthcare workforce as the foundation for patient safety. BMJ Qual Saf. 2024;33(8):483–6. doi: 10.1136/bmjqs-2024-017236 38653549

[70] Bakker AB, Demerouti E, Sanz-Vergel AI. Burnout and work engagement: The JD–R approach. Annu Rev Organ Psychol Organ Behav. 2014;1(1):389–411.

[71] Fiabane E, Giorgi I, Sguazzin C, Argentero P. Work engagement and occupational stress in nurses and other healthcare workers: the role of organisational and personal factors. J Clin Nurs. 2013;22(17–18):2614–24. doi: 10.1111/jocn.12084 23551268

[72] Lepistö S, Alanen S, Aalto P, Järvinen P, Leino K, Mattila E, et al. Healthcare professionals’ work engagement in Finnish university hospitals. Scand J Caring Sci. 2018;32(2):979–86. doi: 10.1111/scs.12538 28994454

[73] Han S. Nurses’ job crafting, work engagement, and well-being: a path analysis. BMC Nurs. 2023;22(1):405. doi: 10.1186/s12912-023-01573-6 37904210 PMC10614409

[74] Zhang Q, Dong G, Meng W, Chen Z, Cao Y, Zhang M. Perceived Stress and Psychological Impact Among Healthcare Workers at a Tertiaty Hospital in China During the COVID-19 Outbreak: The Moderating Role of Resilience and Social Support. Front Psychiatry. 2022;12:570971. doi: 10.3389/fpsyt.2021.570971 35281206 PMC8904916

[75] Alnazly E, Khraisat OM, Al-Bashaireh AM, Bryant CL. Anxiety, depression, stress, fear and social support during COVID-19 pandemic among Jordanian healthcare workers. PLoS One. 2021;16(3):e0247679. doi: 10.1371/journal.pone.0247679 33711026 PMC7954309

[76] Hiles Howard AR, Parris S, Hall JS, Call CD, Razuri EB, Purvis KB, et al. An examination of the relationships between professional quality of life, adverse childhood experiences, resilience, and work environment in a sample of human service providers. Children and Youth Services Review. 2015;57:141–8. doi: 10.1016/j.childyouth.2015.08.003

[77] Theorell T, Hammarström A, Aronsson G, Träskman Bendz L, Grape T, Hogstedt C, et al. A systematic review including meta-analysis of work environment and depressive symptoms. BMC Public Health. 2015;15:738. doi: 10.1186/s12889-015-1954-4 26232123 PMC4522058

[78] Koenig HG. Religion, spirituality, and health: the research and clinical implications. ISRN Psychiatry. 2012;2012:278730. doi: 10.5402/2012/278730 23762764 PMC3671693

