# Supplementary material for: Well-being and quality of life of Kenyan nurses, midwives and community health volunteers: Measurement properties and correlates of the BBQ and WHO-5
Source: PLOS Glob Public Health. 2025 Dec 2;5(12):e0005510. doi: 10.1371/journal.pgph.0005510 (PMC12671763; doi:10.1371/journal.pgph.0005510)
Supplement: S1 Table — (DOCX) [file pgph.0005510.s001.docx]

**S1 Table. Score distribution, alpha if item is deleted, and factor loadings of scale items across study groups.**

|  | **Community Health Volunteers** | | | | **Nurses and midwives** | | | |
| --- | --- | --- | --- | --- | --- | --- | --- | --- |
|  | Mean (SD) | Min - Max | α if Item is deleted | Standardized factor loadings | Mean (SD) | Min - Max | α if Item is deleted | Standardized factor loadings |
| **Brunnsviken Brief Quality of life scale (BBQ)** |  |  |  |  |  |  |  |  |
| 1. You are satisfied with your leisure time: you have the opportunity to do what you want in order to relax and enjoy yourself | 2.77 (0.90) | 0 - 4 | 0.80 | 0.36 | 2.08 (1.23) | 0 - 4 | 0.74 | 0.26 |
| 2. Your leisure time is important for your quality of life. | 3.16 (0.61) | 0 - 4 | 0.79 | 0.50 | 3.35 (0.64) | 0 - 4 | 0.72 | 0.37 |
| 3. You are satisfied with how you view your life: You know what means a lot to you, what you believe in, and what you want to do with your life. | 3.09 (0.71) | 0 - 4 | 0.78 | 0.55 | 3.12 (0.85) | 0 - 4 | 0.70 | 0.50 |
| 4. How you view your life is important for your quality of life | 3.14 (0.60) | 0 - 4 | 0.78 | 0.56 | 3.36 (0.59) | 0 - 4 | 0.70 | 0.55 |
| 5. You are satisfied with opportunities to be creative: to get to use your imagination in your everyday life, in a hobby, on the job, or in your studies. | 3.08 (0.71) | 0 - 4 | 0.78 | 0.54 | 2.94 (0.85) | 0 - 4 | 0.70 | 0.45 |
| 6. Being able to be creative is important for your quality of life | 3.16 (0.54) | 0 - 4 | 0.79 | 0.52 | 3.30 (0.58) | 0 - 4 | 0.71 | 0.49 |
| 7. You are satisfied with your learning: You have the opportunity and desire to learn new, exciting things and skills that interest you. | 3.26 (0.67) | 0 - 4 | 0.78 | 0.56 | 3.14 (0.89) | 0 - 4 | 0.71 | 0.42 |
| 8. Learning is important for your quality of life | 3.31 (0.55) | 0 - 4 | 0.78 | 0.59 | 3.50 (0.56) | 0 - 4 | 0.71 | 0.50 |
| 9. You are satisfied with friends and friendship: You have friends that you associate with and who support you (as many friends as you want and need) | 2.93 (0.79) | 0 - 4 | 0.79 | 0.39 | 2.80 (0.88) | 0 - 4 | 0.71 | 0.35 |
| 10. Friends and friendship are important for your quality of life | 3.06 (0.67) | 0 - 4 | 0.79 | 0.41 | 3.13 (0.73) | 0 - 4 | 0.71 | 0.37 |
| 11. You are satisfied with yourself as a person: You like and respect yourself | 3.33 (0.59) | 0 - 4 | 0.78 | 0.56 | 3.40 (0.63) | 0 - 4 | 0.70 | 0.55 |
| 12. Your satisfaction with yourself as a person is important for your quality of life | 3.28 (0.57) | 0 - 4 | 0.78 | 0.56 | 3.49 (0.55) | 0 - 4 | 0.71 | 0.55 |
| **WHO-5 Item Wellbeing Index** |  |  |  |  |  |  |  |  |
| 1. You have felt cheerful and in good spirits | 3.42 (1.67) | 0 - 5 | 0.73 | 0.65 | 3.34 (1.47) | 0 - 5 | 0.76 | 0.63 |
| 2. You have felt calm and relaxed | 3.14 (1.69) | 0 - 5 | 0.71 | 0.71 | 3.06 (1.44) | 0 - 5 | 0.73 | 0.75 |
| 3. You have felt active and vigorous | 3.25 (1.66) | 0 - 5 | 0.72 | 0.67 | 2.91 (1.49) | 0 - 5 | 0.75 | 0.64 |
| 4. You have woken up feeling fresh and rested | 2.97 (1.72) | 0 - 5 | 0.73 | 0.64 | 2.74 (1.52) | 0 - 5 | 0.75 | 0.65 |
| 5. Your daily life has been filled with things that interest you | 2.61 (1.75) | 0 - 5 | 0.76 | 0.52 | 2.63 (1.51) | 0 - 5 | 0.76 | 0.61 |
