# Supplementary material for: Well-being and quality of life of Kenyan nurses, midwives and community health volunteers: Measurement properties and correlates of the BBQ and WHO-5
Source: PLOS Glob Public Health. 2025 Dec 2;5(12):e0005510. doi: 10.1371/journal.pgph.0005510 (PMC12671763; doi:10.1371/journal.pgph.0005510)
Supplement: S2 Table — (DOCX) [file pgph.0005510.s002.docx]

**S2 Table. Univariate linear regression estimates indicating the association between the total scores of Brunnsviken Brief Quality of life scale and WHO-5 Well-being scale with predictors**

|  | **Community Health Volunteers** | | | | **Nurses/midwives** | | | |
| --- | --- | --- | --- | --- | --- | --- | --- | --- |
|  | **Brunnsviken Brief Quality of life** | | **WHO-5 Well-being** | | **Brunnsviken Brief Quality of life** | | **WHO-5 Well-being** | |
|  | **ß (95% CI)** | **P value** | **ß (95% CI)** | **P value** | **ß (95% CI)** | **P value** | **ß (95% CI)** | **P value** |
| **Socio demographic characteristics** |  |  |  |  |  |  |  |  |
| **Sex** |  |  |  |  |  |  |  |  |
| Male | *Reference* |  |  |  |  |  |  |  |
| Female | **-1.53 (-2.76, -0.31)** | **0.014** | **-1.13 (-1.67, -0.60)** | **< .001** | 0.39 (-0.97,1.75) | 0.574 | -0.38 (-0.88, 0.13) | 0.142 |
| **Age** | -0.02 (-0.07, 0.04) | 0.502 | **0.02 (-0.01, 0.04)** | **< .001** | 0.05 (-0.01, 0.12) | 0.124 | **0.03 (0.01, 0.06)** | **0.007** |
| **Education level** |  |  |  |  |  |  |  |  |
| BSc nursing | *Reference* |  |  |  |  |  |  |  |
| Nursing/midwifery (certificate) | - | - | - | - | -0.86 (-4.60, 2.88) | 0.653 | **2.28 (0.91, 3.66)** | **0.001** |
| Nursing/midwifery (Diploma) | - | - | - | - | -0.71 (-2.46, 1.03) | 0.424 | 0.44 (-0.20, 1.08) | 0.180 |
| MSc nursing | - | - | - | - | 4.61 (-0.54, 9.76) | 0.079 | 1.27 (-0.63, 3.16) | 0.191 |
| Other | - | - | - | - | -1.23 (-4.99, 2.53) | 0.522 | -1.00 (-2.38, 0.39) | 0.157 |
| **Marital status** |  |  |  |  |  |  |  |  |
| Single | *Reference* |  |  |  |  |  |  |  |
| Married | 0.39 (-1.62, 2.39) | 0.706 | -0.34 (-1.21, 0.54) | 0.453 | -0.52 (-1.89, 0.85) | 0.456 | 0.02 (-0.48, 0.53) | 0.932 |
| Divorced/Separated | 1.41 (-2.57, 5.38) | 0.488 | -0.84 (-2.57, 0.90) | 0.344 | 4.31 (-1.27, 9.89) | 0.130 | **2.14 (0.08, 4.21)** | **0.042** |
| Widowed/Widower | -2.17 (-5.09, 0.75) | 0.145 | -0.99 (-2.26, 0.29) | 0.129 | -5.54 (-11.68, 0.60) | 0.077 | -1.34 (-3.6, 0.93) | 0.248 |
| **Psychosocial support from religion** |  |  |  |  |  |  |  |  |
| Yes | *Reference* |  |  |  |  |  |  |  |
| No | **-1.42 (-2.66, -0.18)** | **0.025** | -0.01 (-0.56, 0.53) | 0.959 | **-3.54 (-5.00, -2.07)** | **<.001** | **-0.82 (-1.36, -0.28)** | **0.003** |
| Health Facility |  |  |  |  |  |  |  |  |
| Public | *Reference* |  |  |  |  |  |  |  |
| Private | **-** | **-** | - | - | 0.36 (-1.07, 1.79) | 0.624 | 0.23 (-0.29, 0.76) | 0.386 |
| Faith based organization | **-** | **-** | - | - | 1.73 (-0.85, 4.30) | 0.189 | 0.30 (-0.65, 1.25) | 0.533 |
| Others | **-** | **-** | - | - | 2.90 (-1.53, 7.32) | 0.199 | 0.65 (-0.98, 2.29) | 0.433 |
| **Work duration** |  |  |  |  |  |  |  |  |
| < 10 years | *Reference* |  |  |  |  |  |  |  |
| 11 – 20 years |  |  |  |  | 0.39 (-1.55, 2.33) | 0.692 | 0.10 (-0.62, 0.81) | 0.793 |
| 21 - 30 years | -0.51 (-1.67, 0.64) | 0.382 |  |  | 3.15 (0.83, 5.46) | 0.008 | **1.15 (0.30, 2.00)** | **0.008** |
| 31- 40 years |  |  |  |  | 2.14 (-1.57, 5.84) | 0.259 | **2.16 (0.79, 3.52)** | **0.002** |
| >40 years |  |  |  |  | 7.96 (-5.03, 20.96) | 0.230 | 2.33 (-2.46, 7.12) | 0.341 |
| **Working hours/day** |  |  |  |  |  |  |  |  |
| < 8 | *Reference* |  |  |  |  |  |  |  |
| 8 - 11 | -0.42 (-3.09, 2.25) | 0.756 | -0.62 (-1.78, 0.55) | 0.299 | 1.38 (-0.85, 3.62) | 0.225 | -0.48 (-1.30, 0.35) | 0.259 |
| 12 -16 | 0.03 (-5.63, 5.69) | 0.991 | 0.08 (-2.39, 2.55) | 0.951 | 1.07 (-1.42, 3.55) | 0.401 | **-1.06 (-1.98, -0.14)** | **0.024** |
| > 16 | -4.00 (-13.20, 5.21) | 0.395 | 0.80 (-3.22, 4.82) | 0.697 | -1.13 (-7.66, 5.40) | 0.735 | -1.89 (-4.30, 0.52) | 0.125 |
| **Change in working hours during COVID-19** |  |  |  |  |  |  |  |  |
| Decreased | *Reference* |  |  |  |  |  |  |  |
| Increased | 0.78 (-0.54, 2.10) | 0.245 | 0.07 (-0.51, 0.65) | 0.813 | -1.74 (-3.84, 0.36) | 0.104 | **-0.86 (-1.64, -0.09)** | **0.030** |
| Remained the same | 0.47 (-1.40, 2.35) | 0.621 | 0.44 (-0.38, 1.26) | 0.293 | -0.92 (-2.94, 1.10) | 0.374 | -0.03 (-0.77, 0.72) | 0.945 |
| **Directly exposed to COVID-19** |  |  |  |  |  |  |  |  |
| Yes | *Reference* |  |  |  |  |  |  |  |
| No | -0.56 (-2.07, 0.95) | 0.464 | **0.86 (0.20, 1.51)** | **0.011** | 0.22 (-1.21, 1.66) | 0.760 | **1.56 (1.04, 2.09)** | **<.001** |
| **Tested positive for COVID-19** |  |  |  |  |  |  |  |  |
| Yes | *Reference* |  |  |  |  |  |  |  |
| No | 2.35 (-2.10, 6.80) | 0.301 | 0.84 (-1.10, 2.79) | 0.394 | -0.62 (-2.47, 1.22) | 0.508 | 1.25 (0.57, 1.93) | <.001 |
| **Psychological health** |  |  |  |  |  |  |  |  |
| Resilience | **0.71 (0.49, 0.92)** | **< 0.001** | **0.21 (0.12, 0.31)** | **< 0.001** | **0.95 (0.78, 1.13)** | **<.001** | **0.44 (0.37, 0.50)** | **<.001** |
| Burnout | **-1.01 (-1.18, -0.84)** | **< 0.001** | **-0.43 (-0.51, -0.36)** | **< 0.001** | **-0.86 (-0.99, -0.73)** | **<.001** | **-0.49 (-0.54, -0.45)** | **<.001** |
| Stigmatization Survey | -0.06 (-0.12, 0.00) | 0.072 | **-0.07 (-0.10, -0.05)** | **< 0.001** | **-0.11 (-0.17, -0.04)** | **0.001** | **-0.11 (-0.14, -0.09)** | **<.001** |
| Work Engagement | **0.09 (0.02, 0.15)** | **0.009** | **0.12 (0.09, 0.15)** | **< 0.001** | **0.36 (0.29, 0.43)** | **<.001** | **0.16 (0.14, 0.19)** | **<.001** |
| Social Support | **0.46 (0.39, 0.53)** | **< 0.001** | **0.14 (0.11, 0.18)** | **< 0.001** | **0.66 (0.59, 0.73)** | **<.001** | **0.16 (0.14, 0.19)** | **<.001** |
| Attitude towards seeking psychosocial support | **0.53 (0.39, 0.67)** | **< 0.001** | **0.07 (0.01, 0.13)** | **0.020** | 0.07 (-0.12, 0.26) | 0.474 | **-0.11 (-0.18, -0.04)** | **0.003** |
| Post-traumatic stress disorder | -0.01 (-0.34, 0.32) | 0.959 | **-0.41 (-0.55, -0.27)** | **< 0.001** | **-0.83 (-1.19, -0.47)** | **<.001** | **-0.68 (-0.81, -0.55)** | **<.001** |
| **Health system domain** |  |  |  |  |  |  |  |  |
| Individual capacity and motivation | **1.61 (1.41, 1.81)** | **< 0.001** | **0.33 (0.24, 0.42)** | **< 0.001** | **1.42 (1.24, 1.59)** | **<.001** | **0.50 (0.44, 0.57)** | **<.001** |
| Job factors | **0.81 (0.62, 1.00)** | **< 0.001** | **0.47 (0.39, 0.56)** | **< 0.001** | **1.17 (0.99, 1.34)** | **<.001** | **0.57 (0.51, 0.64)** | **<.001** |
| Work environment | **0.36 (0.27, 0.45)** | **< 0.001** | **0.17 (0.13, 0.21)** | **< 0.001** | **0.43 (0.36, 0.50)** | **<.001** | **0.15 (0.13, 0.18)** | **<.001** |
| Management and leadership | **2.04 (1.74, 2.33)** | **< 0.001** | **0.31 (0.18, 0.44)** | **< 0.001** | **0.72 (0.61, 0.83)** | **<.001** | **0.28 (0.24 ,0.32)** | **<.001** |
| Structural support | **0.73 (0.62, 0.84)** | **< 0.001** | **0.22 (0.17, 0.27)** | **< 0.001** | **0.70 (0.60, 0.79)** | **<.001** | **0.26 (0.22, 0.29)** | **<.001** |
| Perceptions of health system capacity | **1.31 (1.06, 1.55)** | **< 0.001** | **0.46 (0.35, 0.57)** | **< 0.001** | **0.69 (0.46, 0.91)** | **<.001** | **0.17 (0.09, 0.25)** | **<.001** |
